# Self-rated health and general procrastination in nurses: a cross-sectional study

**DOI:** 10.11604/pamj.2020.36.254.23720

**Published:** 2020-08-06

**Authors:** Mahdi Basirimoghadam, Forough Rafii, Abbas Ebadi

**Affiliations:** 1 Nursing Care Research Center, Iran university of Medical Sciences, Tehran, Iran,; 2 Behavioral Sciences Research Center, Life style institute, Baqiyatallah University of Medical Sciences, Tehran, Iran,; 3 Nursing Faculty, Baqiyatallah University of Medical Sciences, Tehran, Iran

**Keywords:** Health status, subjective health, procrastination, nurses

## Abstract

**Introduction:**

nurses are responsible for taking care of the health of the general public. Nurses´ own health is among the important factors affecting the quality of patient care. Self-Rated Health (SRH) is one of the indicators used extensively in health research for the assessment of the health status of individuals. The present study was conducted to evaluate self-rated health and its relationship with general procrastination in nurses.

**Methods:**

the present cross-sectional study was conducted in 2019 on 305 Iranian nurses selected by stratified random sampling. The relationship of self-rated health with procrastination was determined using an ordinal logistic regression analysis after adjustments for personal and occupational factors.

**Results:**

self-rated health was poor/bad in 11.3% of the nurses, fair in 23.7%, good in 34.3% and excellent in 30.7%. After adjustments for personal and occupational factors, a significant relationship was observed between procrastination and self-rated health (OR=0.95; 95%CI 0.92, 0.98).

**Conclusion:**

the results showed an unfavorable health status in nurses. Given the significant relationship between procrastination and poor self-rated health in nurses, it is essential to consider this relationship for improving nurses´ health.

## Introduction

Nursing is a challenging profession that requires physical and emotional resilience. Nurses are a vital workforce for the public health [1], and the community needs their 24-hour critical services [2]. Nurses´ physical and mental health are factors that determine their work efficiency and are associated with the quality of their performance in patient care; in addition, nurses´ good health has extensive consequences for the community. Nurses cannot meet the patients´ needs unless they attend to their own health and feel good about their subjective health [3]. Self-Rated Health (SRH) is a subjective health assessment indicator in health research that has a wide range of applications [4, 5] and is also known as self-perceived health, self-assessed health and subjective health [4]. This indicator is a cost-effective method for measuring the health status that is regarded by the WHO and Euro-REVES as one of the best health measurement indicators at personal and social levels [4, 6]. SRH evaluates the individual´s general health using one question. This simple universal question provides a summary of the individual´s perception of his general health status [7], and despite being a subjective health assessment criterion, it matches the objective health status [4, 8] and predicts mortality [4]. Since SRH is a subjective health assessment criterion, it can be affected by the individual´s mood, and procrastination (unnecessary delay of an intended action despite being aware of the negative consequences of this delay) is associated with several negative mood states, such as anxiety, depression, shame and guilt [9].

Studies have demonstrated the relationship of perceived health with procrastination in students and adults [10, 11], but the relationship between SRH and procrastination has not yet been determined in nurses. Although a study examined the relationship between health-related procrastination and self-rated health in nurses in 2019 [12], it did not consider the relationship between general procrastination (procrastination in daily tasks) and health. Therefore, the present study investigates the relationship between health-related procrastination (procrastination in health tasks such as exercise, diet, etc.) and nurses´ self-rated health, and it is clear that procrastination in performing health duties is connected to health. Furthermore, personal and occupational factors affect both SRH and procrastination [7, 13-15], and the relationship between SRH and procrastination is still unknown even after adjusting for personal and occupational factors. Moreover, there are very few studies in Iran on the health status of Iranian nurses as providers of healthcare. The present study was therefore conducted to evaluate SRH and its relationship with procrastination in nurses after adjustment for personal and occupational factors.

## Methods

**Study design and participants:** the present cross-sectional study was conducted from September 2018 to June 2019 on 305 nurses. Since SRH and its associated factors are different among nurses in metropolises and small towns, the study setting was two hospitals in a metropolis and two in a small town in Iran. The study inclusion criteria for the nurses were having a bachelor´s degree or higher in nursing, a minimum of one year of nursing experience, and not being on a long leave during the data collection process, and the nurses unwilling to take part or who had an experience of severe stress in the last six months (such as divorce, bankruptcy or death of a close relative) were excluded. Stratified random sampling was used, and each hospital was allocated a set quota, and a list of the nurses´ names (with a bachelor´s degree and higher) was obtained from the hospital nursing office, and those with less than one year of work experience or on a long leave were excluded from the list, and the required samples were selected from the list using a simple random method. The required sample size was estimated based on the prevalence of poor SRH, which has been assumed to be 38% among nurses [16]. For a 95% chance of the sample estimate being only 6% above or below this assumed prevalence (38 ± 6%), a sample size of 252 was needed. The sample size was increased by 20% to account for non-response and missing data. The aim was therefore to recruit approximately 305 nurses.

**Data collection:** the nurses filled out the questionnaire on personal and occupational factors, general procrastination and SRH in their workplace. The questionnaire was distributed among them at the start of their shift, and they were asked to complete and return it by the end of the shift.

**Self-rated health:** the most common and universally-accepted measure of health is the SRH, which asks the question of “How do you judge your own general state of health?” which is answered on a 5-point Likert scale (excellent, good, fair, poor, bad) [5]. In the present study, a small number of the nurses rated their SRH badly, and so the “poor” and “bad” rates were merged in the “poor” category, and a 4-point scale was used for the analysis. SRH has a good construct validity [17] and a moderate test-retest reliability [18].

**General procrastination:** it was assessed using the Tuckman procrastination scale, which has 16 items with answers based on a 4-point Likert scale, from “totally disagree” to “totally agree”. A response of “totally disagree” is given 1 point, “disagree” 2 points, “agree” 3 points and “totally agree” 4 points. Four items of this scale are scored in reverse. The minimum score in this scale is 16 and the maximum 64, and higher scores indicate higher procrastination. A standard deviation in excess of the mean is taken to indicate a high procrastinator while a standard deviation less than the mean indicates a low procrastinator. This scale was developed in 1991 by Tuckman *et al*. with a reported Cronbach´s alpha of 0.86 [19]. The validity and reliability of the questionnaire have been confirmed in Iran and its Cronbach´s alpha has been reported as 0.74 [20]. The Cronbach alpha of this scale was calculated as 0.84 in the present study.

**Personal factors:** they included age (in year), gender (female, male), marital status (single, married), education (bachelor´s degree, master´s degree or higher) and underlying diseases, which included diabetes, hypertension, and heart, renal, pulmonary, and gastrointestinal diseases and were assessed by asking one question from the nurses. The nurses were able to choose more than one option, and there was an “other” option to choose and state any other underlying diseases they had. Since only a few nurses had an underlying disease, this question was entered into the analysis as “Underlying disease: “Yes” or “No”.

**Occupational factors:** they included hospital of service (metropolis or town), ward of service (internal, surgery, ICU, pediatrics and emergency), work shift (morning, evening, night, rotational), position (nurse or nursing manager), type of employment (permanent or temporary), work experience (less than 10 years, 10-20 years, over 20 years) and financial satisfaction. The nurses´ financial satisfaction was assessed using one question, i.e. “How satisfied are you with your financial status?”, which was scored based on a 5-point Likert scale of “very little”, “little”, “moderate”, “much” and “very much”. Because of the small number of nurses choosing the “very little” and “very much” categories, these two were merged with the “little” and “much” categories, respectively, thus creating a 3-point scale (“little”, “moderate” and “much”) for the analysis step.

**Ethical considerations:** the present study was conducted with permission from the ethics committee of Iran University of Medical Sciences (code: IR.IUMS.REC 1396.31510). Written consent was obtained from all the participants. The study objectives and voluntary nature of participation were stated at the top of the questionnaire.

**Data analysis:** one of the nurses did not participate in the study due to her heavy workload, and one due to separation from her husband in the last six months. Three questionnaires were discarded because of missing data in excess of 20%, and the analysis was ultimately carried out for 300 nurses. Data were analyzed in SPSS-14.5 (SPSS, Inc., Chicago, Illinois) using descriptive statistics (absolute and relative frequencies) to describe the study variables. First, the distribution of the variables was assessed in the entire sample. Then, the SRH-related factors were identified by bivariate analysis (the Chi-square test). Finally, the variables with p<0.2 were analyzed using the ordinal logistic regression. It should be noted that nurses´ work experience was not included in the ordinal logistic regression model due to its strong correlation with age. The odds ratio and 95% confidence interval were also determined for an excellent SRH. The odds ratio and 95% confidence interval are measured in the attempt to exponentiate the parameter estimates. Microsoft Excel was used for extracting the results of the ordinal logistic regression using an estimation formula for measuring the odds ratio and finding out which variable has a significant effect on the outcome and the significance of that effect for the outcome. The formula used in Excel was “EXP (beta)” for calculating the odds ratio and “EXP (lower bound)” and “EXP (upper bound)” for calculating the 95% confidence interval. p<0.05 was considered statistically significant in a two-tailed test.

## Results

Table 1 presents the distribution of all the variables in the analysis for the entire sample and SRH. SRH was poor/bad in 11.3% of the nurses, fair in 23.7%, good in 34.3% and excellent in 30.7%. A total of 11% of the nurses were high, 76.3% moderate and 12.7% low procrastinators. More than half of the nurses (50.7%) were under the age of 30 years and the majority were female (72.1%) and married (68.7%). A total of 10% of the nurses had a master´s degree or PhD and 90% had a bachelor´s degree. A total of 11.3% had at least one underlying disease. The majority (65.5%) worked in rotating shifts, and fixed evening shifts were the least frequent (7%). A total of 13.7% were nursing managers, 53.7% had temporary work contracts and 46.3% had permanent contracts. A total of 65.7% had less than ten years of work experience, 28.4% had 10 to 20 years and 5.8% had over 20 years. Financial satisfaction was low in 28.5% of the nurses, moderate in 44% and high in 27.5%. SRH had a significant relationship with procrastination (p=0.01), age (p<0.001), gender (p=0.038), marital status (p=0.007), underlying diseases (p<0.001), hospital of service (p=0.005), type of employment (p=0.001), work experience (p=0.012) and financial satisfaction (p<0.001).

**Table 1:** the relationship of self-rated health with the study variables as per the bivariate analyses

Variable	Self-rated health				Total sample	Result
	Bad/Poor	Fair	Good	Excellent		
Total N (%)	34(11.3)	71(23.7)	103(34.3)	92(30.7)	300(100)	
**General procrastination N (%)**						χ2=16.72 df=6 p=0.01
Low	2(5.9)	5(7.0)	14(13.6)	17(18.5)	38(12.7)	
Moderate	23(67.6)	57(80.3)	83(80.6)	66(71.7)	229(76.3)	
High	9(26.5)	9(12.7)	6(5.8)	9(9.8)	33(11.0)	
**Age N (%)**						χ2=25.76 df=6 p<0.001
Less than 30 years	17(51.5)	25(36.2)	43(42.6)	64(70.3)	149(50.7)	
30-40 years	12(36.4)	33(47.8)	42(41.6)	25(27.5)	112(38.1)	
Over 40 years	4(12.1)	11(15.9)	16(15.8)	2(2.2)	33(11.2)	
**Gender N (%)**						χ2=8.44 df=3 p=0.038
Female	29(85.3)	54(76.1)	75(74.3)	57(62.0)	215(72.1)	
Male	5(14.7)	17(23.9)	26(25.7)	35(38.0)	83(27.9)	
**Marital status N (%)**						χ2=12.06 df=3 p=0.007
Married	21(61.8)	58(81.7)	74(71.8)	53(57.6)	206(68.7)	
Single	13(38.2)	13(18.3)	29(28.2)	39(42.4)	94(31.3)	
**Education N (%)**						χ2=5.13 df=3 p=0.16
Master&PhD	0(0.0)	7(9.9)	14(13.6)	9(9.8)	30(10.0)	
Bachelor	33(100.0)	64(90.1)	89(86.4)	83(90.2)	269(90.0)	
**Underlying disease N (%)**						χ2=18.54 df=3 p<0.001
Yes	24(70.6)	59(83.1)	96(93.2)	87(94.6)	266(88.7)	
No	10(29.4)	12(16.9)	7(6.8)	5(5.4)	34(11.3)	
**Hospital of service N (%)**						χ2=12.88 df=3 p=0.005
Metropolis	20(58.8)	37(52.1)	35(34.0)	30(32.6)	122(40.7)	
Town	14(41.2)	34(47.9)	68(66.0)	62(67.4)	178(59.3)	
**Ward of service N (%)**						χ2=18.59 df=12 p=0.099
Internal	11(32.4)	10(14.9)	29(29.9)	29(32.2)	79(27.4)	
Surgery	8(23.5)	22(32.8)	21(21.6)	20(22.2)	71(24.7)	
Intensive	9(26.5)	22(32.8)	30(30.9)	24(26.7)	85(29.5)	
Pediatrics	4(11.8)	8(11.9)	8(8.2)	2(2.2)	22(7.6)	
Emergency	2(5.9)	5(7.5)	9(9.3)	15(16.7)	31(10.8)	
**Shift N (%)**						χ2=6.23 df=9 p=0.72
Morning	7(20.6)	11(15.5)	23(22.4)	13(14.1)	54(18.0)	
Evening	3(8.8)	6(8.5)	6(5.8)	6(6.5)	21(7.0)	
Night	5(14.7)	8(11.3)	6(5.8)	10(10.9)	29(9.7)	
Rotational	19(55.9)	46(64.8)	68(66.0)	63(68.5)	196(65.3)	
**Position N (%)**						χ2=4.04 df=3 p=0.26
Nurse Non manager	30(88.2)	61(85.9)	84(81.6)	84(91.3)	259(86.3)	
Nurse Manager	4(11.8)	10(14.1)	19(18.4)	8(8.7)	41(13.7)	
**Type of employment N (%)**						χ2=15.85 df=3 p=0.001
Permanent	16(47.1)	43(60.6)	52(50.5)	28(30.4)	139(46.3)	
Temporary	18(52.9)	28(39.4)	51(49.5)	64(69.6)	161(53.7)	
**Work experience N (%)**						χ2=16.38 df=6 p=0.012
Less than 10 years	20(62.5)	36(52.9)	62(61.4)	74(81.3)	192(65.8)	
10-20 years	10(31.2)	27(39.7)	31(30.7)	15(16.5)	83(28.4)	
Over 20 years	2(6.2)	5(7.4)	8(7.9)	2(2.2)	17(5.8)	
**Financial satisfaction N (%)**						χ2=34.25 df=6 p<0.001
Low	16(48.5)	30(42.3)	25(24.3)	14(15.4)	85(28.5)	
Moderate	13(39.4)	34(47.9)	45(43.7)	39(42.9)	131(44.0)	
High	4(12.1)	7(9.9)	33(32.0)	38(41.8)	82(27.5)	

Table 2 shows the results of the ordinal logistic regression analysis. After adjusting for the personal and occupational factors, a significant relationship was observed between procrastination and SRH (p=0.003). The odds of being placed in the “high SRH” category reduced by 5% per unit of increase in the nurses´ procrastination (OR=0.95; 95%CI 0.92, 0.98). Moreover, age (p=0.003), education (p=0.039), underlying diseases (p=0.026) and high (p<0.001) and moderate (p=0.009) financial satisfaction were significant predictors of SRH. The odds of being placed in the “high SRH” category reduced by 7% per each year of aging (OR=0.93; 95%CI 0.88, 0.97). The odds of being placed in the “high SRH” category were 2.5 times higher in the nurses with a master´s degree or PhD than the nurses with a bachelor´s degree (OR=2.46; 95%CI 1.05, 5.81) and 2.38 times higher in the nurses without underlying diseases compared to those with diseases (OR=2.38, 95%CI 1.11, 5.10). Moreover, the odds of being placed in the “high SRH” category were 6.82 times higher in the nurses with high financial satisfaction (OR=6.82; 95%CI 3.45, 13.48) and twice higher in those with moderate satisfaction (OR=2.07; 95%CI 1.20, 3.58) compared to the nurses with low financial satisfaction.

**Table 2:** the results of the ordinal logistic regression analysis

variable	In(OR)	p	95% CI
General Procrastination	-0.047	0.003	-0.078-0.016
Age	-0.076	0.003	-0.127-0.026
**Gender**			
Male	0.588	0.052	-0.006-1.182
Female	Ref	Ref	Ref
**Underlying Disease**			
No	0.867	0.026	0.106-1.629
Yes	Ref	Ref	Ref
**Education**			
Master&PhD	0.902	0.039	0.045-1.760
Bachelor	Ref	Ref	Ref
**Marital Status**			
Single	0.254	0.368	-0.299-0.806
Married	Ref	Ref	Ref
**Financial Satisfaction**			
High	1.920	<0.001	1.239-2.601
Moderate	0.728	0.009	0.180-1.277
Low	Ref	Ref	Ref
**Type of Employment**			
Permanent	0.147	0.671	-0.532-0.826
Temporary	Ref	Ref	Ref
**Ward of Service**			
Internal	-0.120	0.787	-0.992-0.751
Surgery	-0.495	0.272	-1.379-0.388
Intensive	-0.103	0.814	-0.958-0.753
Pediatrics	-0.931	0.094	-2.019-0.158
Emergency	Ref	Ref	Ref

Excellent coded as reference category for self-rated health.

## Discussion

According to the results, SRH was reported as poor or bad in 11.3% of the nurses, fair in 23.7%, good in 34.3% and excellent in 30.7%. The frequency of poor SRH is different in different geographic locations. In a study conducted in Brazil, 65.8% of the female nurses and 65.5% of the male nurses had a good SRH while 34.2% of the female nurses and 34.5% of the male nurses had a poor SRH [13]. In another study on Brazilian nurses, 77.6% had a good SRH and 22.4% a poor SRH [21]. In a study conducted in Iran on women from Sanandaj, a good SRH was reported in 62.32% and a poor SRH in 37.68% [16]. A study conducted in Germany reported a poor SRH in 8.5% of the geriatric nurses, 9.2% of the general nurses, and 20.7% of the early childhood education nurses, while the rest had good, very good and excellent SRH [22]. In a study conducted on nurses at hospitals in northwestern Greece, 10.2% had excellent SRH, 31.4% good, 38.5% fair, 18.1% poor and 1.7% very poor SRH [14]. In a study conducted in the US, the health status was reported as excellent by 17% of the participants, very good by 32%, good by 34%, and fair or poor by 17% [18]. The disparity in the results may be due to differences in factors such as financial status, social support and also age.

The present findings showed a low procrastination in 12.7% of the nurses, moderate in 76.3% and high in 11%. In a study conducted on nursing managers and midwifery nursing personnel in one of the hospitals of Iran, 10.9% of the participants had very low, 70.5% had low, 17.1% had moderate, and 1.5% had high procrastination [23]. The results of a study conducted on students showed that none of the participants considered themselves non-procrastinators, 21% did not have serious procrastination, 67% were procrastinators and 12% were serious procrastinators [24]. The results of another study showed that 39% of graduate students and 53% of high school students and undergraduate students always or nearly always procrastinated in doing their academic assignments [19]. The disparity in the results could be attributed to the different prevalence rates of procrastination in different cultures and countries, as the results of a study conducted on the students of a Nigerian nursing school and an Egyptian nursing school showed a high level of academic procrastination in 29.6% of the Egyptian students and a low level in 33.3% of them, while 5.6% of the Nigerian students had a high level of procrastination and 78.8% a low level [25]. People´s cultural values and backgrounds may be effective in their choice of applying or avoiding challenging assignments and also on their interpretation of procrastinating behaviors, and can thus affect the prevalence of procrastination.

A significant relationship was observed between procrastination and SRH in the present study. The odds of being placed in higher SRH categories reduced as procrastination increased. Previous studies have reported a relationship between procrastination and poor perceived health [10, 26]. A possible explanation may be that procrastination leads to unnecessary stress in the individual, and by affecting the immune system, stress can negatively affect health too. Among demographic characteristics, age, education level and underlying diseases had a significant relationship with SRH, but sex and marital status did not have a significant relationship with SRH. In agreement with the results of other studies reporting an inverse relationship between age and SRH [7, 27, 28], the odds of being placed in higher SRH categories reduced with age in the present study too. Jylha [29] calls the relationship between age and SRH as a universal phenomenon. Tigani *et al*. [30] & Meng *et al*. [4] showed that physical functioning reduces with aging, and this reduced functioning can negatively affect SRH.

No significant differences were found between the male and female nurses in terms of SRH. Meanwhile, there are conflicting results about the effect of gender on SRH. A number of studies have identified gender as one of the key predictors of SRH and found that women more commonly report a poor health status than men [14, 31]. Some other studies, however, have not identified gender as a key predictor of SRH [7, 13, 27]. The relationship between gender and SRH requires further studies. Since the majority of nurses working in hospitals are female (72.1% in the present study), such a low male to female ratio makes it difficult to assess the relationship between gender and SRH. The odds of being placed in higher SRH categories were significantly higher in nurses without underlying diseases than in those with diseases. This finding agrees with the results obtained in the study by Haseen *et al*. [7], who identified underlying diseases as one of the main determinants of SRH. The results of a study conducted in Iran showed a significant negative relationship between disease and SRH. Wu *et al*. [32] showed that all diseases are associated with a poor SRH. Meng *et al*. [4] reported disease status as the main predictor of SRH and showed that SRH can reflect the prevalence of chronic diseases, and the present findings, too, support the assumption that SRH can be a reflection of the prevalence of chronic diseases. The odds of being placed in higher SRH categories were greater in nurses with master´s degrees or PhDs compared to nurses with bachelor´s degrees, which agrees with the results obtained by Haseen *et al*. [7] and Mendoza-Romero *et al*. [28]. This finding can be explained by noting that nurses with master´s degrees or PhDs have a greater knowledge of diseases and their consequences, which can enhance their SRH.

In contrast with a study conducted on Thai older adults, which reported a higher SRH in married older adults compared to single ones [7], no significant differences were found between the marital status and nurses´ SRH, but this finding agrees with the results of studies conducted on nurses. In a study conducted on Greek nurses [14] and also a study on Brazilian nurses [13], no significant differences were found in SRH between the single and the married nurses. This disparity in results can be attributed to the fact that married older adults have higher social and family support than single older adults and therefore have a higher SRH; however, this is not the case for nurses, as single working nurses have social and family support too, and marital status was therefore not a significant predictor of SRH in the present study and the studies conducted on nurses in Greece and Brazil. Among the occupational factors, only financial status was significantly related with SRH. The odds of being placed in higher SRH categories were significantly higher in nurses with high or moderate financial satisfaction compared to those with low financial satisfaction. Previous studies have also identified financial status as one of the key variables in SRH [28, 31, 33]. A favorable financial status can affect SRH by affecting the ability to meet one´s basic needs, participation in the community, enjoying life and getting relief from concerns about life emergencies and unexpected disease-related costs.

In agreement with a study conducted on nurses in Greece [14], no significant relationships were found between SRH and variables such as work shift, position and ward of service in nurses in the present study. One of the strengths of the present study was its multi-center setting (both metropolitan and small town hospitals). The study limitations included its cross-sectional design, which does not allow for the determination of the causal relationship between the studied variables. According to our findings, it cannot be concluded that poor SRH causes procrastination in nurses or procrastination leads to SRH in nurses. Therefore, it is recommended that longitudinal studies be conducted. Another limitation was its self-reporting data, which can lead to information measurement bias.

## Conclusion

The present findings showed that, as healthcare providers, nurses themselves have an unfavorable health status. Furthermore, the present study findings highlighted the relationship between procrastination and SRH. Age, underlying diseases, education and financial satisfaction were other factors related with SRH. It is necessary to consider the relationship of these factors with SRH for improving nurses´ health.

### What is known about this topic

- Studies have demonstrated the relationship of perceived health with procrastination in students and adult, but the relationship between self-rated health and procrastination has not yet been determined in nurses;
- Personal and occupational factors affect both self-rated health and procrastination, and the relationship between self-rated health and procrastination is still unknown even after adjusting for personal and occupational factors.

### What this study adds

- After adjustments for personal and occupational factors, a significant relationship was observed between procrastination and SRH;
- Age, underlying diseases, education and financial satisfaction were other factors related with SRH.
